# Using Indoor Air Quality Monitoring in 6 Counties to Change Policy in North Carolina

**Published:** 2009-06-15

**Authors:** Scott Proescholdbell, Julea Steiner, Adam O. Goldstein, Sally Herndon Malek

**Affiliations:** Head, Injury Epidemiology and Surveillance Unit, Injury and Violence Prevention Branch, Division of Public Health, North Carolina Department of Health and Human Services. At the time that this article was written, Mr Proescholdbell was the director of Surveillance and Evaluation for the Tobacco Prevention and Control Branch with the North Carolina Department of Health and Human Services, Raleigh, North Carolina.; University of North Carolina, Chapel Hill, North Carolina; University of North Carolina, Chapel Hill, North Carolina; North Carolina Department of Health and Human Services, Raleigh, North Carolina

## Abstract

**Introduction:**

Indoor air quality monitoring has become a valuable tool for states wanting to assess levels of particulate matter before and after smoke-free policies are implemented. However, many states face barriers in passing comprehensive smoke-free legislation, making such study comparisons unlikely. We used indoor air monitoring data to educate decision makers about the value of comprehensive smoke-free laws in a state with strong historical ties to tobacco.

**Methods:**

We trained teams in 6 counties in North Carolina to monitor air quality in hospitality venues with 1 of 3 possible smoking policy designations: 1) smoke-free, 2) separate smoking and nonsmoking sections (mixed), or 3) smoking allowed in all areas. Teams monitored 152 venues for respirable suspended particles that were less than 2.5 μm in diameter and collected information on venue characteristics. The data were combined and analyzed by venue policy and by county. Our findings were presented to key decision makers, and we then collected information on media publicity about these analyses.

**Results:**

Overall, smoke-free venues had the lowest particulate matter levels (15 µg/m^3^), well below established Environmental Protection Agency standards. Venues with mixed policies and venues that permitted smoking in all areas had particulate matter levels that are considered unhealthy by Environmental Protection Agency standards. The media coverage of our findings included newspaper, radio, and television reports. Findings were also discussed with local health directors, state legislators, and public health advocates.

**Conclusion:**

Study data have been used to quantify particulate matter levels, raise awareness about the dangers of secondhand smoke, build support for evidence-based policies, and promote smoke-free policies among policy makers. The next task is to turn this effort into meaningful policy change that will protect everyone from the harms of secondhand smoke.

## Introduction

Secondhand smoke contains at least 250 chemicals that are toxic or carcinogenic and is itself a human carcinogen (1). Exposure to secondhand smoke causes cardiovascular disease, respiratory illness, and lung cancer, and is responsible for an estimated 40,000 deaths in nonsmokers annually (2,3). Even short-term exposures to secondhand smoke may increase the risk of heart attack (4). The 2006 US Surgeon General's report *The Health Consequences of Involuntary Exposure to Tobacco Smoke* concluded that secondhand smoke causes long-term and short-term health risks, that no levels of secondhand smoke are safe, and that secondhand smoke should be eliminated in all public places (3). The report states, "Eliminating smoking in indoor spaces fully protects nonsmokers from exposure to secondhand smoke. Separating smokers from nonsmokers, cleaning the air, and ventilating buildings cannot eliminate exposures of nonsmokers to secondhand smoke" (3).

The Surgeon General's Report and the *Guide to Community Preventive Services* (3,5,6) state that secondhand smoke exposure and its adverse health effects are preventable. Preventing such exposure is most effectively done by enacting policies requiring smoke-free facilities (3,6). In North Carolina, legislation was introduced that allowed for smoking in "separately ventilated" areas as a way to protect public health. The North Carolina Alliance for Health, the North Carolina Association of Local Health Directors, and the state health department had a goal to make all North Carolina workplaces and public places smoke-free and to exclude exemptions for separately ventilated areas. To that end, they developed criteria for any pending legislation that would meet the public health evidence-based standard outlined by the *Guide to Community Preventive Services* to eliminate all potential secondhand smoke exposures. The challenge was to demonstrate to decision makers why the exemption for separately ventilated areas jeopardized public health.

North Carolina and other states with historic, political, economic, and agricultural ties to tobacco have remained behind the rest of the nation with respect to worker protection from secondhand smoke (7). In North Carolina, 77% of adults report that their workplaces are smoke-free, but differences exist among subpopulations. When examined by subpopulation, among adults with less than a high school education or annual incomes less than $15,000, the proportion with smoke-free workplaces drops substantially (58% among those with less than a high school education and 61% among those with annual incomes less than $15,000). According to Current Population Survey Tobacco Use Supplement data, blue collar (55%) and service industry workers (61%) have less protection from secondhand smoke in their workplaces than do white-collar workers (73%) (8,9).

Support for statewide smoke-free indoor air regulations has been weaker in tobacco farming and manufacturing states than in those with fewer economic ties to tobacco. For example, a 2001 Centers for Disease Control and Prevention (CDC) report assessing policies and attitudes about a ban on smoking in restaurants in 20 states found North Carolina to have the lowest level of support for policy change (10). To move policy forward, health risks must be recognized and quantified. Although many now consider secondhand smoke a serious health hazard, the extent and level of exposure often seemed to be underestimated or misunderstood by decision makers. In the North Carolina General Assembly House Judiciary I Committee hearings on March 21 and April 18, 2006, decision makers raised questions that made it clear they were not aware that simply separating smokers and nonsmokers was not effective in eliminating the health risk.

North Carolina has had a law since 1993 that sets a weak standard at the state level and prevents adoption of stronger ordinances at the local level. This law has been a barrier to comprehensive smoke-free policies at the state level (Smoking in Public Places, General Statute 143-595-601). As of March 2009, 12 states have preemptive state laws prohibiting most new local smoke-free regulations or preventing passage of strong state legislation (11). The strategy in North Carolina has been to reduce these barriers by gaining support for and passing incremental legislation that either bans smoking in certain venues (such as public schools) or permits the passage of smoke-free policies in certain venues (such as public universities). The North Carolina Alliance for Health served as an umbrella group for all tobacco control policy efforts. North Carolina has no regulations on smoking in private workplaces, restaurants and bars, retail stores, or recreational or cultural facilities. Indoor air monitoring has become a tool used by many states to demonstrate the rapid reduction in harmful particulate matter following the passage of city or statewide smoking bans (12-14). These post-policy analyses have demonstrated the effectiveness of the policy, but few studies to date have used this technology as a way to build support for policy change.

CDC focuses on 4 major goal areas, including eliminating nonsmoker exposure to secondhand smoke (15). Tobacco control advocates in North Carolina thought that indoor air monitoring might be a tool to use in efforts to attain this goal. Indoor air monitoring would not only clarify the effect of existing policies but also raise awareness about the levels of secondhand smoke exposure among workers and the general public, increase advocacy on the need for the state to develop stronger secondhand smoke policies, and ultimately attain the goal of smoke-free workplaces and public places with comprehensive legislation. Air monitoring provides a way to illustrate the high levels of hazardous exposure to secondhand smoke in restaurants and increase public policy debate.

## Methods

### Particulate matter data collection

We used a well-established air monitoring protocol developed by Roswell Park Cancer Institute in Buffalo, New York (16). We trained teams composed of state and county health department personnel and community volunteers to conduct air quality monitoring and additional data collection. Teams collected data from October 2005 through May 2007 from a sample of 152 hospitality venues in 6 of North Carolina's 100 counties. These are 6 of the 8 counties where local health departments were receiving CDC tobacco control funding through the state health department (Table 1).

With the assistance of local health departments and community coalitions to prevent tobacco use, we selected a list of venues for testing. Ideally, tested venues would be popular establishments with varying smoking policies. A convenience sample of these venues was identified to make team monitoring in a single day more efficient (ie, clusters of restaurants that could be monitored back-to-back with limited driving or travel time and technical assistance could be provided by the research team within a limited time)With the assistance of local health departments and community coalitions to prevent tobacco use, we selected a list of venues for testing. Ideally, tested venues would be popular establishments with varying smoking policies. A convenience sample of these venues was identified to make team monitoring in a single day more efficient (ie, clusters of restaurants that could be monitored back-to-back with limited driving or travel time and technical assistance could be provided by the research team within a limited time). On entering the venues, teams assigned the venue a secondhand smoke policy based on written, verbal, or visual evidence. Venues had 1 of 3 possible smoking policy designations: 1) 100% smoke-free, 2) separate smoking and nonsmoking sections (mixed), or 3) smoking allowed in all areas.

Air quality monitoring in this study measured respirable suspended particles (RSPs) that were less than 2.5 μm in diameter, known as particulate matter 2.5 (PM_2.5_). PM_2.5_ is harmful fine particles that are released in substantial amounts from burning cigarettes and are easily inhaled deep into the lungs. PM_2.5_ serves as an accurate proxy for exposure to secondhand smoke and has been associated with pulmonary and cardiovascular disease and death (18).

Air quality was monitored by using the TSI SidePak AM510 Personal Aerosol Monitor (TSI, Inc, Saint Paul, Minnesota). The SidePak uses a built-in sampling pump to draw air through the device, which then measures the real-time concentration of PM_2.5_ in milligrams per cubic meter. Teams calibrated the SidePak for 5 minutes outside most venues to obtain a baseline ambient air quality reading. In some instances the team started the machines immediately before entering a venue. They concealed the monitors in purses or business bags and placed them in a central location on a table, counter, or chair in each venue while testing. Teams acted as normal paying patrons at each venue.

Teams collected observational data in each venue for air monitoring. Data included room dimensions, number of people in the room, number of lit cigarettes, and type of smoking policy. The number of people and number of burning cigarettes in each space were recorded every 15 minutes during data collection, and the average number of people and average number of burning cigarettes were calculated. The volume of each venue was also measured by estimating room length, width, and height, and the cigarette density was calculated by dividing the average number of burning cigarettes by the venue volume.

### Particulate matter data analysis

Data analysis began with a venue-level analysis to calculate room size and number of burning cigarettes standardized per 100 m^3^ using direct observation data. The average concentration of PM_2.5_ and monitoring time were also measured for each venue. The monitor recorded measurements every minute, which we averaged for each venue. We discarded the first and last minute of the logged data, and the remaining data points were averaged to provide concentration of PM_2.5_ in each venue.

Venue data were combined and reanalyzed based on observed policy compliance. In addition, all data were pooled to evaluate particulate matter concentrations for all venues regardless of observed policy compliance (N = 152 sites). Smoker density and room volume were analyzed. Average monitoring time was calculated for each venue.

All air monitoring data were analyzed by using SPSS 14.0 for Windows (SPSS, Inc, Chicago, Illinois).

### Media and advocacy tracking

We asked county and state programs to submit and track any "earned media" (free publicity gained by promoting the study results) and presentations of air monitoring results. We worked with coalition members to create presentations, talking points, and lists of frequently asked questions showing the results for each county. These were to be presented to the public and to media and public policy makers at the state and county level.

## Results

### Particulate matter data

The mean time spent in each venue was 46 minutes (range, 15-129 minutes) excluding outside air measurements before and after entering the venue. The minimum time was set at 30 minutes unless the venue had fewer than 5 patrons. The length of stay beyond the minimum was dependent on the volunteer teams and their expectations for monitoring venues. Longer stays tended to happen in larger, more crowded venues that had longer waits. Because teams were encouraged to act like normal patrons, some stayed for extended times. However, extremes of the range were atypical.

Average particulate matter concentration for all smoke-free locations (n = 45) was 15 µg/m^3^ whereas the average PM_2.5_ concentration in all mixed venues (n = 67) was 67 µg/m^3^. Those venues with no smoking policy (n = 40), allowing smoking in all areas, had the highest PM_2.5_; the average for all smoking venues was 253 µg/m^3^. This value represents 16 times the exposure of the average smoke-free venue and more than 7 times the maximum of 35 µg/m^3^ considered safe by the Environmental Protection Agency (EPA) (19).

**Figure 1:**
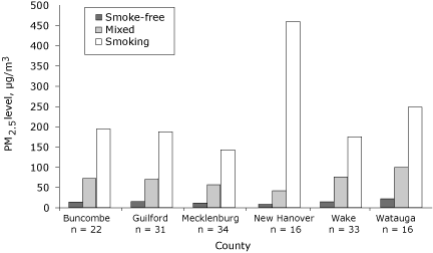
Average levels of respirable suspended particles that are less than 2.5 μm in diameter (PM_2.5_), by county and by secondhand smoke policy designation of the venues tested in the North Carolina Indoor Air Study, 2005-2007. The study teams assigned each venue a secondhand smoke policy based on written, verbal, or visual evidence of either 1) 100% smoke-free, 2) separate smoking and nonsmoking sections (mixed), or 3) smoking allowed in all areas.

Table 2 shows the average PM_2.5_ concentration by county and venue policy designation. When averaged by county, venues with smoking allowed in all areas had substantially higher levels of PM_2.5_ than did those with mixed or smoke-free policies (Table 2). All smoke-free venues were below the EPA standard (Figure 1). All mixed venues had PM_2.5_ levels above the standard. All venues that permitted smoking in all areas had levels above 136 µg/m^3^; the range was from 143 to 459 µg/m^3^. Although venues with mixed policies had elevated levels of PM_2.5_, compared with smoke-free venues no significant differences were noted.

**Table:** 

**Policy**	**PM_2.5_ Level, μg/m^3^ (No. of Venues)**					

	**Buncombe (22)**	**Gilford (31)**	**Mecklenburg (34)**	**New Hanover (16)**	**Wake (33)**	**Watauga (16)**
Smoke-free	14	15	11	8	14	22
Mixed	72	70	56	41	76	99
Smoking	194	187	143	459	175	248

Room volume did not substantially differ among the groups. However, smoker density was much higher in venues that allowed smoking.

### Media and advocacy tracking

Researchers from the North Carolina Tobacco Prevention and Control Branch and the University of North Carolina Tobacco Prevention and Evaluation Program, along with local public health and advocacy partners, were engaged in developing and presenting results to key stakeholders to build support for a sound statewide secondhand smoke policy. Earned media included, in 2 counties, front-page stories of their local results and radio and television coverage.

**Figure 2:**
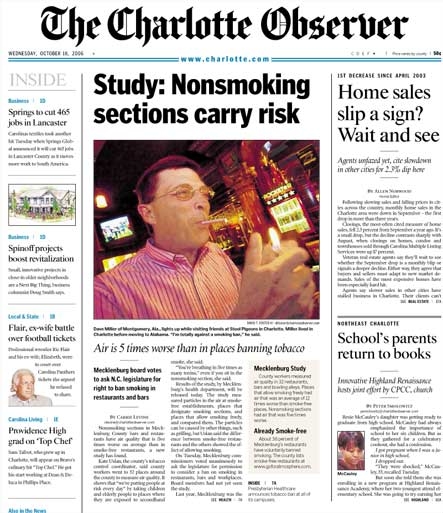
Front page of the *Charlotte Observer*, October 18, 2006, an example of media activity generated by the North Carolina Indoor Air Study, 2005-2007.

In Charlotte, the largest urban center, a front-page story in the *Charlotte Observer *(Figure 2), the largest circulation newspaper in the state, led to 1 editorial, 1 regular columnist column, 2 public policy blogs, and 16 letters to the editor. Following the news release, 500 signatures were added to a petition circulated by the Smokefree Charlotte coalition (later expanded to Smokefree Mecklenburg, the county where Charlotte is located). The local smoke-free restaurants Web site had 10 times the number of visitors than in the previous month. Air monitoring data were presented to public policy makers across Mecklenburg County. The result was that 4 of 6 town councils, 1 city council, and the Board of County Commissioners voted to support local authority to pass regulations on smoking in public places.

Other presentations were made by the North Carolina Tobacco Prevention and Control Branch with local tobacco control partners to 4 local boards of health, the local health director's liaison committee of the state health department, 5 local health directors, and 5 tobacco control coalitions. Presentations were also made to statewide health coalitions such as the North Carolina Alliance for Health and legislative committees such as the Justus-Warren Heart Disease and Stroke Prevention Task Force.

Several state legislators sought secondhand smoke information, and they received the air monitoring data as part of an information package from North Carolina Alliance for Health members. The House majority leader, who had planned to introduce legislation regulating smoking in workplaces in the legislative session, requested and was provided a briefing by the head of the North Carolina Tobacco Prevention and Control Branch. Finally, legislators representing the western North Carolina region requested and were provided a presentation just before the legislative session convened; local health department tobacco control partners gave this presentation. Public health advocates also incorporated the air monitoring findings into their existing presentations on secondhand smoke for use in training and advocacy meetings. The state health department packaged the data and they were given to local health department directors, tobacco control staff, and local partners such as American Heart Association members. The North Carolina Alliance for Health served as a repository and helped facilitate distribution of the data. Most local presentations were made by local county health department staff. As part of the state contract with these local agencies we also made several presentations.

## Discussion

Indoor air monitoring data across North Carolina show that, in the absence of comprehensive public health protections at the state or local level, levels of RSPs remain unacceptably high in multiple hospitality establishments statewide. Restaurants with complete bans on indoor smoking have substantially lower RSPs than do venues with no or minor limitations on smoking. The data show that venues allowing smoking (separate or not) can substantially reduce indoor exposure to secondhand smoke among customers and staff by becoming smoke-free.

The effect of these data is unclear. However, the media coverage combined with several advocacy efforts seemed to have some effect with key stakeholders who had not previously publicly supported secondhand smoke policy restrictions, thus increasing support for statewide secondhand smoke policies. At the state level, the results helped educate policy makers considering the passage of House Bill 259: Act to Prohibit Smoking in Food and Lodging Establishments and State Government Buildings and Allow Local Governments to Prohibit Smoking in Public Places and Places of Employment. The act was narrowly defeated in the North Carolina House (61 to 55), but this bill provided the greatest health protection and was the best showing of support for the policy to date. A previous bill considered by the North Carolina General Assembly in 2005 was considerably weaker in public health terms by creating loopholes for separately ventilated areas and exempting certain venues. Support that might have come from the Senate and the governor's office is unknown as neither ever considered this or other bills, although historically the challenges to such legislation came from the House. These results illustrate that the use of indoor air monitoring has the potential not only to demonstrate effectiveness of policy change but also may play a role in building support for evidence-based policy change.

As of March 2009, only 13 states have enacted 100% smoke-free worksite laws that include restaurants and bars (20). Studies analyzing these policy changes have found significant reductions in secondhand smoke in every location tested (21). The North Carolina findings are consistent with these studies but differ in terms of how the data can be used. In Delaware, RSP levels declined similarly in 8 hospitality venues after state law prohibited smoking there (12). In New York, a study observed declining RSP levels in 20 hospitality venues after a smoking ban was put into place (14). However, previous studies of indoor air quality have largely ignored states with laws pre-empting stronger local controls and only examined changes before and after laws were implemented.

Several studies of the effects of smoking bans suggest that the long-term heath effects could be substantial as a result of these policies (13,22). Some indicate that respiratory health improved rapidly among workers after smoke-free workplace laws went into effect (22,23). A growing number of studies demonstrate reductions in acute myocardial infarctions from 8% to 40% in locations such as Helena, Montana; Pueblo, Colorado; Bowling Green, Ohio; northern Italy; and, most recently, New York State (24-29).

These findings are subject to several limitations. First, the venues chosen for this study may not be representative of all venues in North Carolina or elsewhere. However, we sampled a variety of sizes, types, and locations. Second, secondhand smoke is not the only source of indoor particulate matter. Although ambient particle concentrations and cooking smoke are additional sources of indoor particulate levels, secondhand smoke is the largest contributor to indoor RSP pollution (30). Additionally, air quality was monitored in public service areas where secondhand smoke is the most likely source for concentrations measured. Third, the popularity of hospitality venues and the number of customers varied from venue to venue. Therefore, the level of active smoking in any given area at a given time varied from place to place. For this reason, PM_2.5_ concentrations may not accurately represent actual overall levels but be specific to that time. Fourth, although the testing times per venue were similar to those in prior studies, the testing time might not have been representative of a particular venue because of the range of hours that venues are open. Finally, this was an exploratory study looking at the possible effects of using indoor air monitoring to influence policy change. We have shown this type of research is feasible statewide, to generate publicity and data used in policy debates. We do not know, however, the exact role that the research can and would play with a more rigorous study design. Our work shows that additional research and evaluation are warranted.

Twenty-seven states lack comprehensive smoke-free legislation. Of these, 12 states face tough preemption laws that effectively limit local level and state level change (11). For an investment of $12,000 (4 machines at $3,000 each), we trained local county volunteers to collect data from their communities and then used that data to raise awareness and educate policy makers at both the local and state level. This study design can be readily replicated in all areas that face similar constraints. In cases where statewide laws cannot be easily achieved, air monitoring may be a valuable tool to assist tobacco control advocates in influencing policy change.

Nineteen states (Arizona, Delaware, Florida, Hawaii, Illinois, Iowa, Louisiana, Maryland, Massachusetts, Minnesota, Montana, Nevada, New Jersey, New York, Ohio, Oregon, Rhode Island, Utah, Washington) and Puerto Rico meet the national health objective for 2010 calling for implementation of statewide smoking bans in worksites, which includes hospitality venues (although 4 of those states have bans that do not cover bars). Comprehensive smoking bans will also take effect in Nebraska in June 2009 and in Montana in October 2009 (20). These states account for approximately 45% of the US population. To further reduce the nearly 40,000 deaths among never smokers caused by secondhand smoke each year, similar comprehensive laws are needed in the other 31 states and the District of Columbia.

## Figures and Tables

**Table 1:** Demographics of Participating Counties — North Carolina Indoor Air Study, 2005-2007

**County**	Population (2006 Estimated)^a^	Major City^b^	No. of Venues Tested	Average Time, min^c^
Buncombe	221,320	Asheville	22	44
Guilford	449,078	Greensboro	31	40
Mecklenburg	826,893	Charlotte	34	43
New Hanover	184,120	Wilmington	16	57
Wake	790,007	Raleigh	33	47
Watauga	43,410	Boone	16	55

a Data from the Office of State Budget and Management (17).

b All venues tested were within the major city.

c Based on a grand mean calculation for all venues within a monitored area.

**Table 2:** Average Levels of Respirable Suspended Particles That Are Less Than 2.5 μm in Diameter (PM_2.5_) Among Venues in Participating Counties, by Secondhand Smoke Policy Designation^a^ — North Carolina Indoor Air Study, 2005-2007

County (No. of Venues)	Dates of Monitoring	Average PM_2.5_ Level (μg/m^3^)		

		Smoke-Free	Mixed	Smoking
Buncombe (22)	May 2006	14	72	194
Guilford (31)	April and May 2006	15	70	187
Mecklenburg (34)	January 2006	11	56	143
New Hanover (16)	March 2007	8	41	459
Wake (33)	October 2005, April 2006, February 2007	14	76	175
Watauga (16)	April 2007	22	99	248

a The study teams assigned each venue a secondhand smoke policy based on written, verbal, or visual evidence of either 1) 100% smoke-free, 2) separate smoking and nonsmoking sections (mixed), or 3) smoking allowed in all areas (smoking).
